# Endostatin in 3D Fibrin Hydrogel Scaffolds Promotes Chondrogenic Differentiation in Swine Neonatal Meniscal Cells

**DOI:** 10.3390/biomedicines10102415

**Published:** 2022-09-27

**Authors:** Valentina Rafaela Herrera Millar, Barbara Canciani, Laura Mangiavini, Joel Fernando Soares Filipe, Lucia Aidos, Margherita Pallaoro, Giuseppe Maria Peretti, Paola Pocar, Silvia Clotilde Modina, Alessia Di Giancamillo

**Affiliations:** 1 Department of Biomedical Sciences for Health, University of Milan, Via Mangiagalli 31, 20133 Milan, Italy; 2 IRCCS Istituto Ortopedico Galeazzi, Via Riccardo Galeazzi, 4, 20161 Milano, Italy; 3 Department of Veterinary Medicine and Animal Sciences (DIVAS), University of Milan, Via dell’Università 6, 26900 Lodi, Italy

**Keywords:** endostatin, fibrin hydrogel scaffold, chondrocyte, differentiation, morphology, meniscal tissue engineering

## Abstract

The success of cell-based approaches for the treatment of cartilage or fibro-cartilaginous tissue defects requires an optimal cell source with chondrogenic differentiation ability that maintains its differentiated properties and stability following implantation. For this purpose, the aim of this study was to evaluate the use of endostatin (COL18A1), an anti-angiogenic factor, which is physiologically involved in cell differentiation during meniscus development. Swine neonatal meniscal cells not yet subjected to mechanical stimuli were extracted, cultured in fibrin hydrogel scaffolds, and treated at two different time points (T1 = 9 days and T2 = 21 days) with different concentrations of COL18A1 (10 ng/mL; 100 ng/mL; 200 ng/mL). At the end of the treatments, the scaffolds were examined through biochemical, molecular, and histochemical analyses. The results showed that the higher concentration of COL18A1 promotes a fibro-chondrogenic phenotype and improves cellularity index (DNA content, *p* < 0.001) and cell efficiency (GAGs/DNA ratio, *p* < 0.01) after 21 days. These data are supported by the molecular analysis of collagen type I (COL1A1, a marker of fibrous-like tissue, *p* < 0.001), collagen type II (COL2A1, a marker of cartilaginous-like tissue, *p* < 0.001) and SRY-Box Transcription Factor 9 (SOX9, an early marker of chondrogenicity, *p* < 0.001), as well as by histological analysis (Safranin-O staining), laying the foundations for future studies evaluating the involvement of 3D endostatin hydrogel scaffolds in the differentiation of avascular tissues.

## 1. Introduction

Meniscus repair or regeneration is still an unsolved clinical challenge, mainly due to the morpho-functional peculiarities of this tissue. The menisci are fibro-cartilaginous structures, located in the knee joint and interposed between the femoral condyles and the tibial plateaus. Each knee consists of two menisci, a medial and a lateral one. Microscopically, the menisci are made up of an extracellular matrix (ECM) and cells secreting it.

In mammals, the neonatal meniscus appears fully vascularized, but with advancing age, the inner zone loses its vascularity. For this reason, the inner area of the tissue is called the “white-white zone”, while the outer area, which maintains a certain degree of vascularization in adults, is called the “red-red zone”. The intermediate zone is called the “red-white zone”. Previous studies have verified that the change in the vascularity of the meniscus coincides with a phenotypic change in both the cells and the cellular matrix [1,2,3,4,5].

To date, it is possible to identify changes that occur, at least, at four different levels, although they are all interconnected with each other: (i) the phenotype of cells pass from a fibroblast-like phenotype in the inner zone to a chondrogenic-type phenotype [1,6]; (ii) the composition of the matrix changes with age: it mainly consists of type I collagen in newborns, while in adults, type II collagen is mostly synthesized, especially in the inner zone; moreover, cellularity decreases but cells increase the production of glycosaminoglycans [7]; (iii) the biomechanical properties change, the increase in aggrecan synthesis enhances the compressive strength while the reduction in type I collagen decreases the ability to resist tension forces [8,9,10]; and (iv) the vascularization changes, that is, from completely vascularized tissue at the periphery, and becomes avascularized in the inner zone [1,6,11].

The structure and composition of the meniscus make it particularly resistant to loading forces but with poor regenerative capacity, especially in the avascular area. While a lesion in the red-red zone can undergo repair mechanisms, a white-white lesion is unable to heal spontaneously and correctly [12,13]. To date, various treatments are being studied but meniscal tear repairs are still an active challenge in the field of orthopedics since there are still no treatments available that completely restore functionality to the tissue and prevent osteoarthritic phenomena [14,15,16,17,18,19,20,21,22,23].

Tissue engineering for the meniscus combines engineering with biological sciences in order to improve the functions of a tissue or an organism. It is still difficult to reproduce *in vitro* conditions that physiologically can be found in the knee. First of all, the joint environment is physiologically hypoxic [24,25,26,27,28]. Recent studies published by our research group demonstrated the role that hypoxia has in the differentiation of neonatal meniscal tissues, i.e., it favors the expression of endostatin, an antiangiogenic factor that could be involved in the differentiation of meniscal cells, and favors the deposition of the matrix, especially in the posterior horn [27,28]. Both fibroblasts and fibro-chondrocytes express this protein [1,27] but, in young animals, the highest peak of endostatin is recorded together with phenotypic changes of meniscal cells (from fibroblast-like to fibro-chondrocytes), while lower endostatin expression is found to be associated with decreased vascularity, typical of the adult animal. Therefore, endostatin could play a key role in the differentiation of meniscal cells.

In addition to the joint environment, the three-dimensional supports favor chondrogenic differentiation [29,30,31,32,33]. In fact, it was reported that chondrocytes tend to de-differentiate in monolayer, becoming fibroblast-like cells [34]. This mechanism often occurs after the implantation of chondrocytes in the joint, inducing post-operative fibrosis phenomena [35]. In this context, hydrogels represent a promising prospect. They are semi-solid solutions of water and polymers which gelatinize or precipitate depending on the polymer [36,37]. They have good biocompatibility and mechanical properties, and—being porous structures—they allow the infiltration and survival of cells inside them [36,38,39]. In particular, previous studies demonstrated that fibrin glue hydrogel is able to promote chondrocyte survival and synthetic activity *in vitro* [40,41] and *in vivo* [42,43], leading to the formation of tissue with cartilage-like properties. Moreover, other studies [44] that focused on the role of fibrin glue in delivering chondrocytes into solid scaffolds demonstrated that this gel is able to increase cell seeding efficiency and uniformity promoting a major matrix deposition as a result of an efficient rescue of the chondral phenotype [41].

In view of this evidence, the aim of this study was to evaluate the role that exogenous endostatin administration could have in a hydrogel scaffold seeded with neonatal porcine meniscal cells: more precisely, we analyzed the capacity of pig neonatal cells to differentiate, under diverse endostatin concentrations and culture times, through the evaluation of (1) cellularity, (2) production of glycosaminoglycans, and (3) type I, type II collagen, and SRY-Box Transcription Factor 9 (SOX9) gene expression, in a biocompatible hydrogel fibrin scaffold, in order to lay the basis for studying the influence of angiogenesis factors to promote the regeneration of a meniscal lesion in the avascular area.

## 2. Materials and Methods

### 2.1. Experimental Design

**Figure 1:**
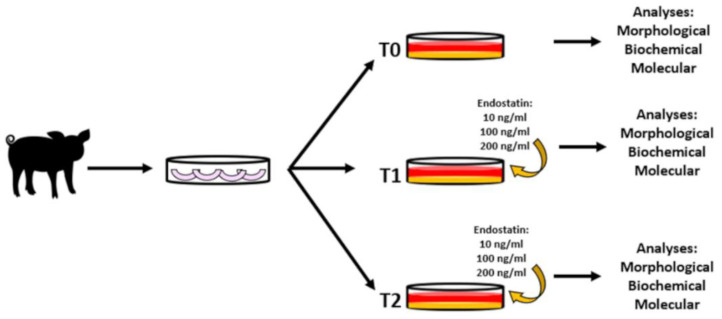
Experimental design. Meniscal cells were isolated and expanded to P3. At this passage, the fibrin glue hydrogel was assembled and the cells were added. Scaffolds were cultured in different concentrations of exogenous endostatin (10 ng/mL, 100 ng/mL, 200 ng/mL) and in negative control conditions by means of no endostatin added. After 9 or 21 days *in vitro*, the scaffolds were collected for morphological, biochemical, and molecular analysis.

Meniscal cells from newborn piglets were isolated to study the basal expression of endostatin. Three experimental times were considered (T0 = starting point [passage 3], T1 = 9 days in culture, T2 = 21 days in culture), as well three different concentrations (10 ng/mL; 100 ng/mL; 200 ng/mL) of exogenous endostatin (#NP_569712, R&D System, Minneapolis, MN, USA) were added to meniscal cell culture of newborn piglets starting at the passage 3 (P3). At the end of each time point, morphological, biochemical and molecular analyses were performed (Figure 1).

### 2.2. Sample Collection

In compliance with the 3Rs principles (Replace, Reduce and Refine) and the Legislative Decree number 26 of 2014, no animals were sacrificed for experimental purposes. Sixty knee joints (n. of animals = 30) were collected from newborn piglets that died under the weight of the mother on a local farm.

### 2.3. Cell Culture

Medial menisci were isolated in aseptic conditions and minced with surgical scissors. The fragmented tissues were digested in Dulbecco’s modified Eagle’s medium (DMEM, Thermo Fisher Scientific, Waltham, MA, USA) containing Collagenase II 3.75 g/mL (Worthington Biochem. Corp., Lakewood, NJ, USA) overnight at 37 °C at 245 rpm on a shaker. The following day, the digest was filtered with 70 µm filters and then centrifuged twice at 350 g for 10 min, re-suspending it in sterile phosphate-buffered saline (PBS, Thermo Fisher Scientific, Waltham, MA, USA). The pellet that was deposited after the second centrifuge was re-suspended in culture medium consisting of DMEM with fetal calf serum (FCS 10%, EuroClone, Pavia, Italy), glutamine (2 mM, Thermo Fisher Scientific, Waltham, MA, USA), antibiotics (100 U/mL, Penicillin–streptomycin, Thermo Fisher Scientific, Waltham, MA, USA, 50 µg/mL Gentamicin sulfate, LONZA, Basel, Switzerland), amphotericin B (0.5 µg/mL, EuroClone, Pavia, Italy), HEPES (10 µM, Thermo Fisher Scientific, Waltham, MA, USA), sodium pyruvate (1 mM, Thermo Fisher Scientific, Waltham, MA, USA), FGF-2 (1 ng/mL, R&D System, Minneapolis, MN, USA), and 1 ng/mL TGF-β1 (R&D System, Minneapolis, MN, USA). Live cells were counted with a hemocytometer chamber and subsequently expanded in culture medium up to passage 3 (P3) in standard culture conditions (21% O_2_, 37 °C, 5% CO_2_).

### 2.4. Immunofluorescence: Basal Expression of Endogenous Markers

By immunofluorescence (IF), basal expression of cell endogenous markers at T0/P3 was studied using the following antibodies: type I and type II collagen (anti-COL1 Millipore, Temecula, CA, USA and anti-COL2 Abcam Laboratories, Cambridge, UK, respectively), SRY-Box Transcription Factor 9 (anti-SOX9, Abcam Laboratories, Cambridge, UK), endostatin (anti-ENDO, Millipore, Temecula, CA, USA) and vascular endothelial growth factor A (anti-VEGFA, am Laboratories, Cambridge, UK). To perform IF, 20.000 cells/cm^2^ at P3 were seeded on matrigel (Corning, NY, USA) in 24-multiwell plate and when they reached 80% of confluence, they were washed with PBS and fixed in cold (−20 °C) methanol (Thermo Fisher Scientific, Waltham, MA, USA) for 10 min. After a further three washes in PBS, the nonspecific sites were blocked with 10% Normal Goat Serum (NGS, Thermo Fisher Scientific, Waltham, MA, USA) in PBS for 30 min. The primary antibodies were diluted 1:50 and a 2 h-incubation at room temperature (RT) was performed. The secondary antibody (Thermo Fisher Scientific, Waltham, MA, USA) was diluted 1:500 in PBS 1% of NGS and incubated for 1 h RT. Wells were washed again in PBS and after incubation with Hoechst’s solution (Thermo Fisher Scientific, Waltham, MA, USA) in PBS for 5 min, the slides were sealed to a microscope slide by fluorescent mounting media (DAKO, Sant Clara, CA, USA). Fluorescent images were captured by Confocal Laser Scanning Microscope (FluoView FV300; Olympus Italia, Milan, Italy).

### 2.5. Scaffold Preparation of Fibrin Glue Hydrogel

Hydrogel scaffolds (HS) were prepared as published by Deponti et al. [43] with some modifications. Briefly, after being trypsinized (0.05% trypsin EDTA, EuroClone, Pavia, Italy) at P3, the cells (80 × 10^6^ cells/mL) were re-suspended in a solution named A containing fibrinogen bovine plasma (113.2 mg/mL, Aigma-Aldrich, St. Louis, MA, USA), aprotinin (3.24 mg/mL, Sigma-Aldrich, St. Louis, MA, USA), tranexamic acid (30 mg/mL, Sigma-Aldrich, St. Louis, MA, USA) in PBS. The scaffolds were assembled in a 96-well plate: first, 150 µL of solution A was placed in a well, secondly, 150 µL of solution B (thrombin, 13.7 mg/mL in PBS, Millipore, Temecula, CA, USA) was added. The scaffolds polymerized after 30 min under a sterile hood at room temperature, and then they were removed from wells and immersed for 9 days or for 21 days in different concentrations of exogenous endostatin (R&D System, MN, USA) dissolved in culture medium: (i) 10 ng/mL (E10), (ii) 100 ng/mL (E100), (iii) 200 ng/mL (E200), (iv) no endostatin added as negative control (UNT). Four replicates for each treatment and time points were maintained *in vitro*, changing culture medium twice a week. At the end of each time point, samples were stored at −80 °C for biochemical and molecular analyses or fixed in 4% buffered formalin (Bio-Optica, Milan, Italy) for morphological analysis.

### 2.6. Morphological Analyses: Safranin-O Staining

After fixation, HS were dehydrated in an increasing scale of ethanol (70%, 95%, 100%, Bio-Optica, Milan, Italy), clarified in xylene (Bio-Optica, Milan, Italy) and embedded in paraffin (VWR International Srl, Milan, Italy) and 4 µm sections were cut. Standard stain protocol was used to evaluate morphology and GAGs deposition. Briefly, the slices were hydrated to water using xylene and a decreasing ethanol scale (100%, 95%, 70%, 50%). The sections were stained by 1 min incubation of hematoxylin (Bio-Optica, Milan, Italy), followed by 10 min of washing in tap water. Then, the sections were incubated for 5 min in a 0.05% solution of Fast Green (FCF, Sigma-Aldrich, St. Louis, MA, USA) in distilled water, and quickly rinsed in 1% acetic acid (Sigma-Aldrich, St. Louis, MA, USA) solution in distilled water. A 5 min counterstain was performed with a 0.1% Safranin-O solution (Sigma-Aldrich, St. Louis, MA, USA). Finally, slices were dehydrated in alcohol solution (95%, 100%) and clarified with xylene.

### 2.7. Biochemical Analyses

To quantify the DNA content and GAGs production, HS were digested in a papain solution (Sigma-Aldrich, St. Louis, MA, USA) as described by Aidos et al. (2022). After digestion, the DNA content was estimated using Quant-iT Picogreen dsDNA Assay Kit (Molecular Probes, Eugene, OR, USA) according to the kit instructions, while the production of GAGs was estimated using the 1,9-dimethylmethylene blue due binding assay (Polysciences Europe GmbH, Hirschberg an der Bergstrasse, Germany).

### 2.8. Molecular Analyses

Total RNA was extracted using RNeasy Mini Kit (Qiagen, Hilden, Germany) and was quantified with Nanodrop 8000 (Thermo Fisher Scientific, Waltham, MA, USA). ImProm II Reverse Transcription System (Promega, Milan, Italy) was used to RNA reversely transcribe. The primers (Eurofins Genomics, Ebersberg, Germany) listed in Table 1 [27,28] were used to amplify type I and type II collagen (COL1A1 and COL2A1) and SRY-Box Transcription Factor 9 (SOX9) genes by Real-Time PCR (7500 Fast Real-Time PCR System, Applied Biosystems) with PowerUp SYBR master mix (Thermo Fisher Scientific, Waltham, MA, USA). Β-actin (ACT) gene was used as reference. Three reaction steps were performed: (1) holding stage at 95 °C for 3 min; (2) 40 cycles of 98 °C for 15 s and then 60 °C for 1 min; (3) melting curve was used to exclude unspecific amplifications. ΔCt measurements were determined by cycle threshold (Ct) of the target gene minus the Ct of reference gene for each sample.

### 2.9. Statistical Analyses

Statistical analyses were performed with GraphPad Prism software. Two-way ANOVA, considering the treatment and the time as variables, was used to analyze data, followed by Tukey’s multiple comparisons test. For greater clarity, graphs were split in two in order to illustrate as clearly as possible the effects of time and the effects of the treatment. In the figures, data were expressed as mean ± the standard error of the mean. Differences were considered significant when *p* < 0.05.

## 3. Results

### 3.1. Immunofluorescence: Basal Expression of Endogenous Markers

**Figure 2:**
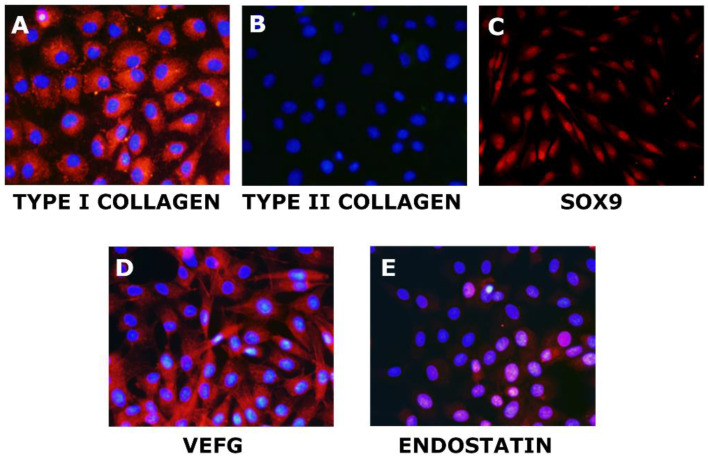
Basal expression of endogenous markers in meniscal cells: positive staining for type I Collagen (Red staining—(**A**)), no signal for type II Collagen (**B**), positive staining for SOX9 (Red staining —(**C**)), positive signal for VEGF (Red staining—(**D**)), positive staining for endostatin (pink staining at a nuclear level—(**E**)). The blue signal in all images is the Hoechst staining of cell nuclei.

Chondrogenic markers were used to analyze the basal expression of meniscal cells at P3. Meniscal cells were able to express type I collagen in the cell cytoplasm (Figure 2A). They appeared negative for type II collagen (Figure 2B), but they were positive for SOX9 (Figure 2C). The investigation of vascular-related markers revealed that meniscal cells expressed both cytoplasmatic VEGFA (Figure 2D) and weak nuclear endostatin (Figure 2E).

### 3.2. Scaffolds Analyses

#### 3.2.1. Safranin-O Staining: GAGs Deposition

**Figure 3:**
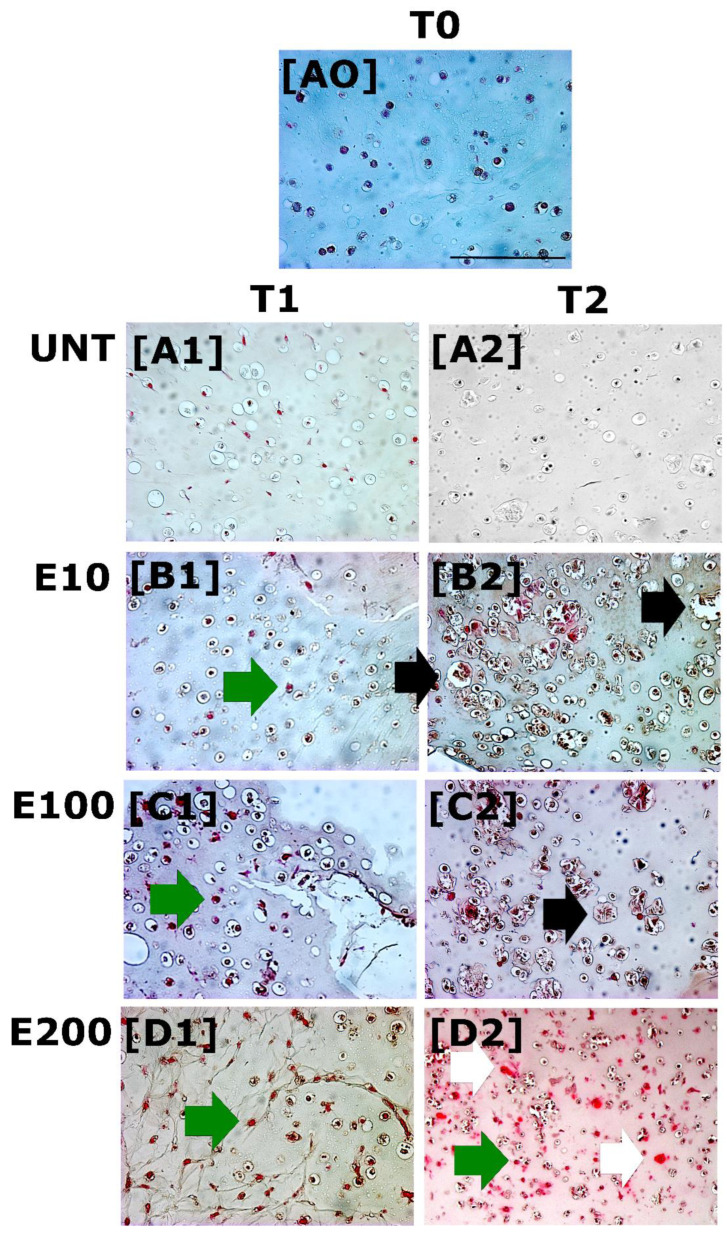
GAGs deposition was evaluated by Safranin-O staining at T0 (d = 0, (**A0**)), T1 (d = 9 days *in vitro*, (**A1**–**D1**)) and T2 (d = 21 day *in vitro*, (**A2**–**D2**)). (**A0**) UNT group at T0; (**A1**) UNT group at T1; (**A2**) UNT group at T2; (**B1**) E10 at T1; (**B2**) E10 at T2; (**C1**) E100 at T1; (**C2**) E100 at T2; (**D1**) E200 at T1; (**D2**) E200 at T2. Green, white and black arrows indicate cellular magenta spots, the territorial matrix and isogenous groups, respectively. Scale bar: 50 µm.

At the end of each experimental time, HS were collected for Safranin-O staining, which is specific for GAGs deposition where the cartilage-like tissue appears magenta colored. T0 (Figure 3A0) and negative control (Figure 3A1,A2) appeared completely negative for Safranin-O staining. At T1, a weak positivity for Safranin-O staining was observed in all endostatin-treated groups as cellular magenta spots (Figure 3B1–D1, green arrows). After 21 days (T2) of endostatin-treated culture, GAGs deposition increased especially in E200, showing a more evident staining in the territorial matrix (Figure 3D2, white arrows) as well as cellular positivity (Figure 3D2, green arrows). In the T2 endostatin-treated groups, E10 and E100, unlike E200, a larger isogenous group in cell cultures (Figure 3B2–C2, black arrows) was present.

#### 3.2.2. Biochemical Analysis: DNA and GAGs Content

**Figure 4:**
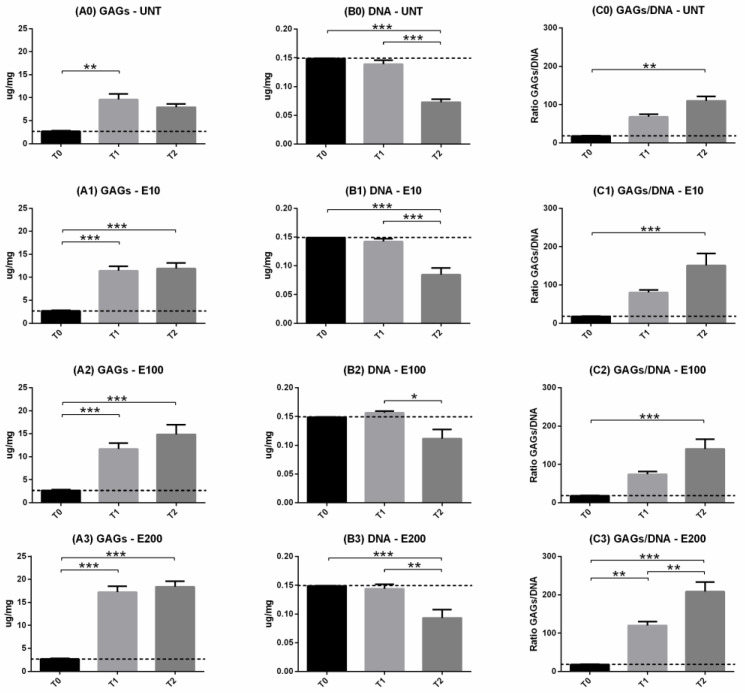
Biochemical analysis: GAGs (**A0**–**A3**) and DNA (**B0**–**B3**) content and their ratio GAG/DNA (**C0**–**C3**). Trends over time (9 days = T1 and 21 days = T2) were illustrated for each treatment. Two-way ANOVA and Tukey’s multiple comparisons test were performed. N = 4; * = *p* < 0.05; ** = *p* < 0.01; *** = *p* < 0.001. Data were expressed as mean ± S.E.M. The dashed black line was the expression level corresponding to T0.

**Figure 5:**
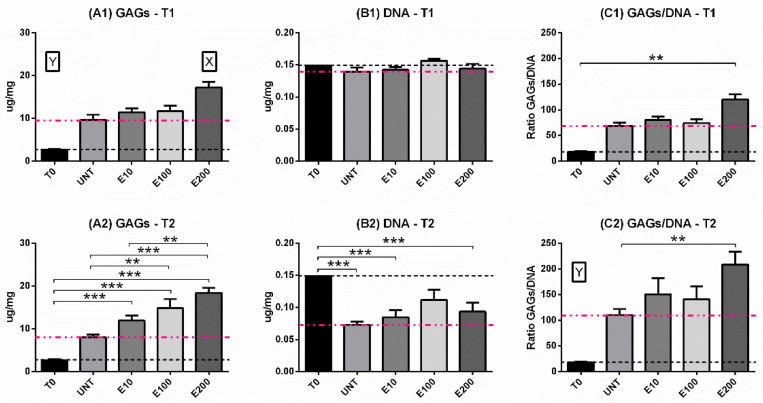
Biochemical analysis: GAGs (**A1**,**A2**) and DNA (**B1**,**B2**) content and their ratio GAG/DNA (**C1**,**C2**). Comparison between the time points (9 days = T1 and 21 days= T2) for all treatments. Two-way ANOVA and Tukey’s multiple comparisons test were performed. N = 4; ** = *p* < 0.01; *** = *p* < 0.001. Z, Y and X were used when the treatment was significantly different (0.001, 0.01 and 0.05, respectively) from all other treatments and to T0 at the considered time point. Data were expressed as mean ± S.E.M. The dashed black line is the expression level corresponding to T0, while the pink one corresponds to the expression level of the UNT group.

GAG production, DNA content, and their ratio (GAG/DNA) were evaluated as productivity, viability, and efficiency indexes, respectively. In all endostatin treatments, cells improved their ability to produce GAGs matrix over time (*p* < 0.001) (Figure 4A0–A3). At T1, E200 significantly increased GAGs production compared to all other experimental groups (*p* < 0.05; Figure 5A1). After 21 days, both E100 and E200 showed higher matrix production than both negative controls (*p* < 0.01) and T0 (*p* < 0.001) (Figure 5A2), while no significant differences were observed between the E10 and UNT groups (Figure 5A2). DNA content decreased over time in all treatments (*p* < 0.05) (Figure 4B0–B3). No differences were observed after 9 days (Figure 5B1) compared to T0. Although all T2 treatments showed a significant decrease in terms of viability when compared to T0 and T1 (Figure 4B0–B3), E100 T2 did not show significant differences compared to T0 and T1 (Figure 5B2).

Concerning the GAGs/DNA ratio, it increased over time in all treatments (*p* < 0.01) (Figure 4C0–C3). In addition, T2 of all endostatin treatment groups showed higher efficiency compared to T0 (*p* < 0.01) (Figure 4C0–C3). Furthermore, T1 E200 was significantly higher compared to T0 (*p* < 0.01) (Figure 5C1) and UNT (*p* < 0.01) (Figure 5C2).

#### 3.2.3. Gene Expression: COL1, COL2, SOX9

**Figure 6:**
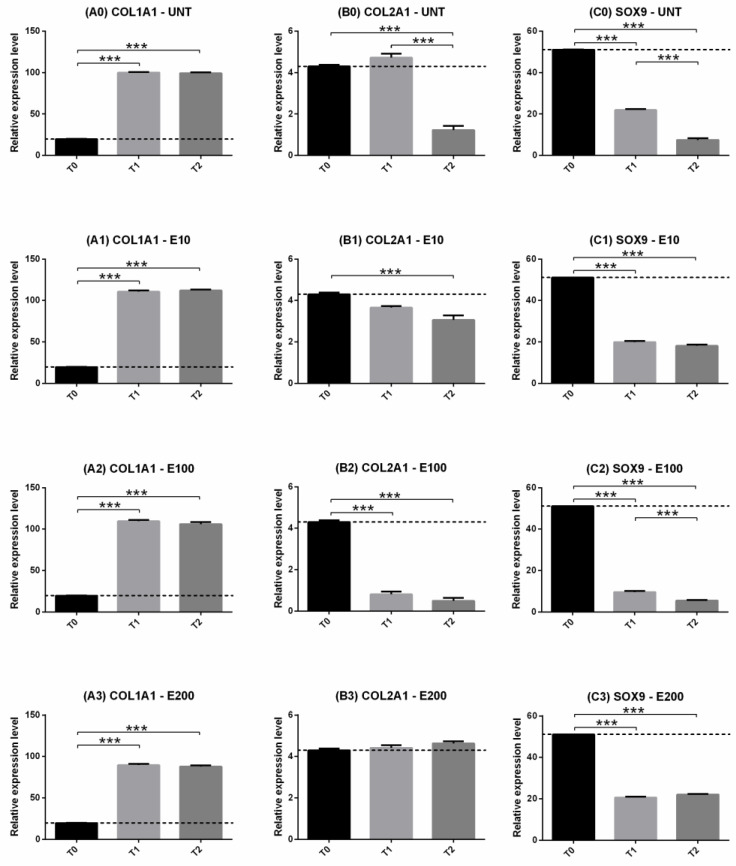
Gene expression the day of seeding (T0), after 9 days (T1) and after 21 days (T2) in culture: COL1A1 (**A0**–**A3**), COL2A1 (**B0**–**B3**), SOX9 (**C0**–**C3**). Two-way ANOVA and Tukey’s multiple comparisons test were performed. N = 4; *** = *p* < 0.001. Data were expressed as mean ± S.E.M. The dashed black line was the expression level corresponding to T0.

**Figure 7:**
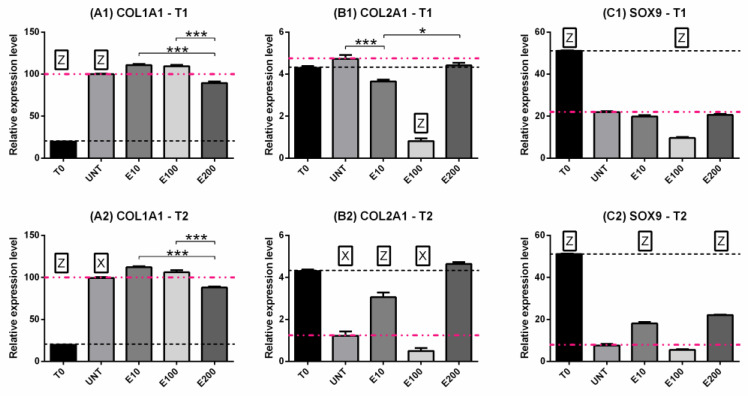
Gene expression: COL1A1 (**A1**,**A2**), COL2A1 (**B1**,**B2**), SOX9 (**C1**,**C2**). Comparison between the time points T1 (**A1**–**C1**) and T2 (**A2**–**C2**) for all treatments. Two-way ANOVA and Tukey’s multiple comparisons test were performed. N = 4; * = *p* < 0.05; *** = *p* < 0.001. Z, Y and X were used when the treatment was significantly different (0.001, 0.01 and 0.05, respectively) from all other treatments and to T0 at the considered time point. Data were expressed as mean ± S.E.M. The dashed black line was the expression level corresponding to T0, while the pink one corresponds to the expression level of the UNT group.

Type I collagen expression significantly increased over time (*p* < 0.001) in all treatments, but no differences were observed between T1 and T2 (Figure 6A0–A3). E10 and E100 increased COL1A1 gene expression at both T1 (Figure 7A1) and T2 (Figure 7A2) compared to UNT (*p* < 0.05) or T0 (*p* < 0.001). Instead, treatment with E200 induced a lower upregulation of the gene than the UNT group compared to T0 (Figure 7A1–A2).

Type II collagen had a variable trend over time according to the treatments (Figure 6B0–B3). UNT, E10 and E100 induced downregulation of the gene over time (Figure 6B0–B2), but E200 maintained always its expression (Figure 6B3). Concerning the treatments, E100 had already induced a decrease in COL2A1 expression after 9 days compared to all other groups (Figure 7B1), but at T2, E10 and E200 were upregulated compared to the UNT groups (Figure 7B2, pink dotted line).

SOX9 decreased its expression in all treatments over time compared to T0 (*p* < 0.001) (Figure 6C0–C3), but in the E200 and E10 groups, no significant differences were observed at T2 compared to T1 (Figure 6C1,C3). After 9 days of treatments, no significant differences were observed in the groups treated with endostatin except for E100 compared to the negative control (*p* < 0.001) (Figure 7C1, pink dotted line); after 21 days, E200 and E10 were able to maintain higher gene expression compared to the UNT group (*p* < 0.001) (Figure 7C2) which, in turn, had a significant downregulation compared to T1 (*p* < 0.001) (Figure 6C0).

## 4. Discussion

One of the goals of tissue engineering is to regenerate and repair meniscal lesions in the inner area of the meniscus, with an adequate cellular source, in the most suitable culture environment and possibly in a material that favors adequate differentiation. Based on our previous works, we believed that another important point to consider could be meniscal vascularization, which varies during growth. For this reason, we developed a protocol using neonatal cells with high differentiation potential, a fibrin hydrogel scaffold and endostatin as a growth factor. Neonatal cells were chosen as their unaltered plasticity makes them particularly sensitive to endostatin stimuli in a suitable *in vitro* culture system: basal expression of these cells revealed the presence of COL1A1, COL2A1, SOX9, and VEGF as they belong to a very vascularized tissue and a weak nuclear expression of endostatin, which is expected to increase with age. This feature is in accordance with the differentiation capacity of meniscal cells which tend to decrease with age [45,46,47]. Furthermore, these results are in line with the previous works published by our research group [1,27,28], while in the literature there are no references to the basal expression of neonatal porcine meniscal cells. Nevertheless, Vanderploeg et al. (2012) evaluated the matrix proteins of young bovine (2–4 years old)—an animal that already walks—by identifying both the expression of type I collagen and type II collagen [48].

For the *in vitro* culture substrate, we adopted a fibrin glue hydrogel previously used for the chondrogenic differentiation of articular chondrocytes [43]. This biopolymeric material exhibits excellent biocompatibility and promotes cell attachment [49]; moreover, it was shown to be an excellent polymer for generating cartilaginous tissue [43,50,51,52,53,54,55,56].

Finally, we decided to add endostatin, one of the most potent inhibitors of angiogenesis [57], as a growth factor in the *in vitro* system. As a result, we verified that endostatin is able to influence the expression of chondrogenic markers such as COL1A1, COL2A1, and SOX9. The idea of verifying the role of endostatin on meniscal cell differentiation stems from observations on menisci of newborn, young and adult pigs, where we observed that tissue maturation was modulated by the endostatin, which is highly expressed in young animals [1,27]. Moreover, Hoberg et al. [58] observed that the endothelial cells of the vessels present in the meniscus produce biological factors, including endostatin, that can contribute to the healing of lesions. The same authors [58] studied the expression of different angiogenic factors in human fibro-chondrocytes upon co-culture with endothelial cells, to investigate the potential role of meniscal repair. They found an increase in endostatin expression by fibro-chondrocytes correlated with a significant reduction of proliferation in endothelial cells, thus suggesting that endostatin may contribute to the poor healing capacities in the less vascularized zone of menisci. Additionally, Pufe et al. [59] published that the spatial and temporal expression of endostatin in the meniscus is important for its development and for the maintenance of avascular zones.

Other authors were interested in anti-angiogenic molecules: Fuji et al. [60] have indeed studied anti-angiogenic factors, such as chondromodulin-I (ChM-I) and endostatin in the meniscus. They revealed that ChM-I was mainly detected in meniscal inner and superficial zones, on the other hand, endostatin distribution was similar between the inner and outer meniscus. The use of anti-angiogenic factors as growth promoters was speculated for ChM-I, which was considered a key regulatory molecule that is involved in angiogenic switching in cartilage during bone formation. The authors suggest that in the avascular zone of cartilage, ChM-I participates in the rapid growth of endochondral bone formation and matrix synthesis [61]. Finally, Klinger et al. [62] observed that ChM-I stabilizes the chondrocyte phenotype by supporting chondrogenesis but inhibiting chondrocyte hypertrophy and endochondral ossification.

Our last consideration was about the concentration of endostatin that we tested: the doses of endostatin reported for meaningful effects *in vitro* and in animal models vary. In a lung cancer model, a dose of 10 mg/kg of endostatin was just as effective in suppressing tumor growth [63]. A concentration of 100 ng/mL was reported to be sufficient to inhibit endothelial cell proliferation *in vitro* [64]. Another *in vitro* investigation [65] showed that endostatin inhibited angiogenic factors in human endothelial cells in a dose-dependent manner, with the dose–response peaking at a concentration of 200 ng/mL. Such a range of responses from the literature drove us to use the range from 10 to 200 ng/mL, revealing that in committed meniscal cells, the higher concentration is the promising one.

Taking into account these interesting results, we observed that high concentrations of endostatin were able to stimulate neonatal meniscal cells in the fibrin glue to produce a huge amount of GAGs compared to all other groups. Furthermore, even if cellularity—expressed as DNA content—decreased, cell efficiency significantly increased in the group with the highest endostatin concentration (E200). It is important to consider that in previous *in vivo*, *ex vivo* and *in vitro* studies, meniscal cellularity normally decreases over time and thus becomes mature tissue, as occurs for hyaline cartilage [1,10,27,28,66,67,68,69,70]. The integrity of chondrocytes or fibro-chondrocytes is critical to maintaining tissue homeostasis. Thus, if endostatin was able to promote the production of one of the major components of the meniscus extracellular matrix, this angiogenic factor could lay a good foundation in meniscal tissue engineering. In more detail, E200 was the concentration that was most promising for cellular efficiency after both 9 days and 21 days in culture. Similarly, Sun et al. [71] demonstrated the promising results of the non-viral delivery of the gene for endostatin to caprine marrow stromal cells (MSCs) via a collagen scaffold: they prepared 21 days of cultured MSC pellets which were revealed to be stained intensely with Safranin-O, demonstrating the high content of GAGs. Morphologically, the cells displayed a rounded shape, typical of the chondrocyte phenotype. The same authors [72] have also studied a collagen scaffold-based cartilaginous construct overexpressing endostatin, using non-viral transfection. Endostatin-plasmid-supplemented collagen scaffolds were seeded with mesenchymal stem cells and human nasal chondrocytes and cultured for around 21 days. They found a higher GAGs density in scaffolds with endostatin-expressing cells and morpho-functional analyses confirmed the presence of GAGs with an intense Safranin-O positive staining. In our study, the trend of genes related to chondrogenesis suggested that lower endostatin concentrations (E10 and E100) increased the type I collagen gene, decreased the type II collagen gene, and the trend of SOX9 was variable, while E200 downregulated the type I collagen compared to the negative control, and it maintained the expression levels of type II collagen, which was instead downregulated in all the other groups as well as SOX9. Hoberg et al. [58] found that the expression of endostatin changed upon co-culture with endothelial cells whereas the expression of all other extracellular matrix proteins (collagen I, II) remained unchanged; on the contrary, in our study, we found an increase in the ECM proteins associated to an increase in the endostatin concentration in the culture medium. These differences could be related to the *in vitro* culture support, as fibrin hydrogel could have improved chondrogenic markers expression according to Adesida et al. [73] due to its three-dimensional structures that could stabilize cartilaginous cells [74].

The correct composition of the extracellular matrix is essential for the regeneration of healthy tissue. Several imaging studies suggest that osteoarthritic menisci have degenerated extracellular matrix and fibers without a regular orientation [75,76,77]. It is assumed that this degeneration could be due to hypertrophic mechanisms affecting the meniscal cells, but to date, there are no studies that validate this theory. When hypertrophy or osteoarthritic phenomena occur, SOX9 expression decreases [78,79]. SOX9 is known to be expressed in progenitor cells but its expression remains high only in chondrogenic differentiation [80,81,82]. As concerns our study, we observed that after 9 days in culture, endostatin-treated fibro-chondrocytes actually showed a SOX9 downregulation but this response was also observable in the untreated group (UNT); hence, it is plausible to hypothesize that this downregulation is not due to the endostatin recombinant protein but rather attributable to the *in vitro* culture conditions. Furthermore, it is interesting to observe how, after 21 days of culture, E200 was able to maintain high SOX9 expression compared to both of the other treatments and to the untreated group, where a further collapse in the gene expression occurs. Further studies are needed in the future to investigate the link between endostatin and hypertrophy and to modify the associated culture environment, precisely matching endostatin and hypoxia.

At this point, we have to face the two main limitations of this research: (1) the static *in vitro* culture and (2) the environmental culture conditions. Static culture definitely cannot mimic what occurs *in vivo* in the knee joint, where at least three different types of forces (compression, traction, sliding) act on the tissue, in addition to the action—albeit minimal—induced by the movement of synovial fluid around the meniscus [10,83]. To mimic these forces, it is essential to have an available bioreactor, and currently, there are no bioreactors capable of mimicking the three forces mentioned above at the same time. Puetzer et al. [84] published the most effective bioreactor that could be used in meniscal tissue engineering but it mimics tension and compression forces [84]. Concerning the environmental conditions of the culture, we used standard culture conditions (37 °C, 5% CO_2_, 21% O_2_) but the joint space is physiologically a hypoxic environment (1–9% O_2_) [24,25,26]. Hence, the ideal culture conditions would require a bioreactor under hypoxic conditions.

## 5. Conclusions

The present work suggests that *in vitro* culture of meniscal cells in a fibrin hydrogel scaffold, in the presence of endostatin, improved the differentiation of fibroblast cells into fibro-chondrocytes. This represents a considerable achievement since one of the limitations facing meniscal tissue engineering is the *in vitro* non-replicable phenotype and thus functions of the tissue due to limited knowledge regarding the biology and the physiology of the tissue. Further studies are required to improve *in vitro* cultures in order to mimic the physiological joint environment in the most suitable way possible.

## Figures and Tables

**Table 1:** Primer sequences used in Real-Time PCR showing the forward and reverse sequences of the chosen primers, with the resulting amplicon length.

Gene	Forward (5′-3′)	Reverse (5′-3′)	Amplicon Size (bp)
COL1A1	CCA ACA AGG CCA AGA AGA AG	ATG GTA CCT GAG GCC GTT CT	64
COL2A1	CAC GGA TGG TCC CAA AGG	ATA CCA GCA GCT CCC CTC T	102
SOX9	CCG GTG CGC GTC AAC	TGC AGG TGC GGG TAC TGAT	119
ACT	CAA GGA GAA GCT CTG CTA CG	AGA GGT CCT TCC TGA TGT CC	245

## Data Availability

Publicly available datasets were analyzed in this study. This data can be found here: https://osf.io/q9xup/?view_only=fe9dd924591e4ed483e249924e226e8c (accessed on 26 July 2022).
